# Realization of Osteolysis, Angiogenesis, Immunosuppression, and Drug Resistance by Extracellular Vesicles: Roles of RNAs and Proteins in Their Cargoes and of Ectonucleotidases of the Immunosuppressive Adenosinergic Noncanonical Pathway in the Bone Marrow Niche of Multiple Myeloma

**DOI:** 10.3390/cancers13122969

**Published:** 2021-06-13

**Authors:** Takashi Watanabe

**Affiliations:** Department of Personalized Cancer Immunotherapy, Mie University Graduate School of Medicine, 2-174, Edobashi, Tsu City 514-8507, Japan; twatanabe@med.mie-u.ac.jp

**Keywords:** multiple myeloma, isatuximab, daratumumab, mesenchymal stem cells, osteoclasts, ectoenzyme, CD38, extracellular vesicle, exosome, microRNA, long noncoding RNA, PIWI-interacting RNA

## Abstract

**Simple Summary:**

Multiple myeloma (MM) is a disease that extensively involves bone, and angiogenesis and immunosuppression are important processes in the development of MM. Proteasome inhibitors and immunomodulatory drugs remarkably improve the survival of MM patients. However, MM is still an incurable disease that rapidly becomes resistant to these drugs. There is robust evidence that extracellular vesicles (EVs) contribute to cancer metastasis. Osteoclasts, in addition to immunosuppressive cells in the bone marrow (BM), are key players in osteolysis and immunosuppression. BM stromal cells and MM cells secrete EVs through which they communicate with each other: EVs, in fact, contain proteins, small RNAs, and long non-coding RNAs that mediate this communication and contribute to angiogenesis, osteolysis, and cancer dissemination and drug resistance. Ectoenzymes are expressed in myeloma cells, osteoclasts, and stromal cells and produce immunosuppressive adenosine. Recently, an antibody targeting CD38, an ectoenzyme, has been shown to improve the survival of patients with MM. Thus, understanding the properties of EV and ectoenzymes will help elucidate key processes of MM development.

**Abstract:**

Angiogenesis and immunosuppression promote multiple myeloma (MM) development, and osteolysis is a primary feature of MM. Although immunomodulatory drugs and proteasome inhibitors (PIs) markedly improve the survival of patients with MM, this disease remains incurable. In the bone marrow niche, a chain of ectoenzymes, including CD38, produce immunosuppressive adenosine, inhibiting T cell proliferation as well as immunosuppressive cells. Therefore, anti-CD38 antibodies targeting myeloma cells have the potential to restore T cell responses to myeloma cells. Meanwhile extracellular vesicles (EVs) containing microRNAs, proteins such as cytokines and chemokines, long noncoding RNAs, and PIWI-interacting RNAs have been shown to act as communication tools in myeloma cell/microenvironment interactions. Via EVs, mesenchymal stem cells allow myeloma cell dissemination and confer PI resistance, whereas myeloma cells promote angiogenesis, myeloid-derived suppressor cell proliferation, and osteoclast differentiation and inhibit osteoblast differentiation. In this review, to understand key processes of MM development involving communication between myeloma cells and other cells in the tumor microenvironment, EV cargo and the non-canonical adenosinergic pathway are introduced, and ectoenzymes and EVs are discussed as potential druggable targets for the treatment of MM patients.

## 1. Introduction

Multiple myeloma (MM) is the second most common hematologic malignancy, characterized by the accumulation of monoclonal neoplastic plasma cells at multiple sites in the bone marrow (BM) [1]. In almost all patients with MM, a premalignant state called monoclonal gammopathy of undetermined significance (MGUS) precedes MM [2,3]. The response rate and overall survival (OS) of patients with MM have improved significantly since the development of novel drugs, including the proteasome inhibitor bortezomib and the immunomodulatory drugs thalidomide and lenalidomide; however, the 6-year OS of patients over 65 years of age is still only 56% [4], and MM remains incurable. Indeed, most patients relapse or become refractory to the abovementioned therapies, suggesting that drug resistance may decrease the efficacy of MM treatments [5].

**Figure 1:**
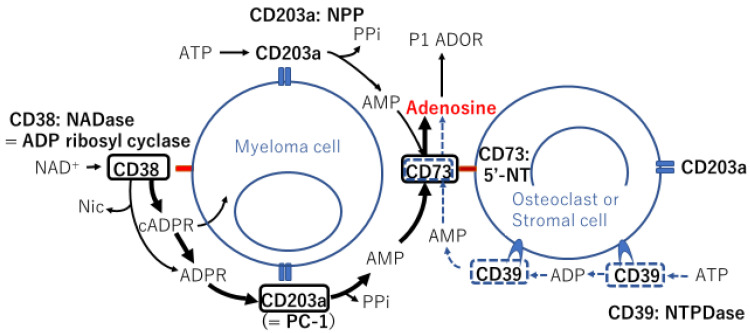
Schematic representation of ectoenzymes expressed on the surface of two different cell types in the bone marrow microenvironment and enzymatic reaction chains with their substrates, involving canonical and noncanonical adenosinergic pathways. Ectoenzymes in black squares and bold arrows indicate the noncanonical pathway, whereas ectoenzymes in blue dotted squares along with dotted arrows indicate the canonical pathway. NADase: nicotinamide adenine dinucleotidase; ADP: adenosine diphosphate; NAD^+^: nicotinamide adenine dinucleotide^+^; Nic: nicotinamide; cADPR: cyclic adenosine diphosphate ribose; ADPR: adenosine diphosphate ribose; PPi: inorganic pyrophosphate; AMP: adenosine monophosphate; ATP: adenosine triphosphate; NPP: nucleotide pyrophosphatase/phosphodiesteras; P1 ADOR: purinergic type 1 adenosine receptor; 5′-NT: 5′-nucleotidase; NTPDase: nucleoside triphosphate diphosphohydrolase.

Furthermore, monoclonal antibodies (mAbs) have been developed for the treatment of MM; for example, the anti-CD38 antibody daratumumab is now a standard first-line treatment [6,7]. Recently, isatuximab, which has a stronger ability to inhibit the ectoenzyme function of CD38 than daratumumab [8], was approved for the treatment of patients with refractory/relapsed MM. CD38 is an ectoenzyme located in the plasma membrane that converts nicotinamide adenine dinucleotide (NAD)^+^ to immunosuppressive adenosine (Figure 1) [9]. Ectoenzymes are excreted in microvesicles (MVs) because of their intrinsic location on the cytoplasmic membrane and may contribute to the progression of MGUS to MM [10], creating an immunosuppressive state in the BMM [11].

The bone marrow microenvironment (BMM) comprises several cell components, including endothelial cells (ECs), osteoclasts, osteoblasts, stromal cells, mesenchymal stem cells (MSCs), myeloid-derived suppressor cells (MDSCs), and regulatory T cells (Tregs). These cells build a niche to support MM cell growth, particularly in the hypoxic state, in which the noncanonical adenosinergic pathway is predominantly facillitated (Figure 1) [12].

Recently, extensive evidence has shown that MM and stromal cells of the BMM use extracellular vesicles (EVs) [11,13,14,15,16,17,18], including exosomes [19,20,21,22,23,24], as a communication tool; MM cells educate MSCs [13,25], and conversely, MSCs contribute to MM cell spreading [20]. Exosomes are a subfraction of EVs, ranging from 60 to 120 nm in size. Two exosome subpopulations have been identified by asymmetric-flow field-flow fractionation, i.e., large exosomes 90–120 nm and small exosomes 60–80 nm [26]. They are actively secreted, contain cell-specific, bioactive molecules, and exert their functions by transferring their cargo to their target cells, by either clathrin-dependent or -independent endocytosis or micropinocytosis [27]. Exosomes arise via an endocytic pathway; early endosomes give rise to the formation of small intraluminal vesicles (ILVs), then ILV-containing structures transform and become multivesicular bodies, a process mainly regulated by a group of endosomal sorting complexes required for transport, and finally the matured vesicles are detached from the late endosomes. In contrast, MVs are formed via the outward budding and fission of the plasma membrane and are 50–1000 nm in size [28].

Exosomes or EVs carry a varied cargo, including lipids, proteins, mRNAs, and microRNAs (miRNAs). Several protein-coding mRNAs have been shown to have natural antisense transcript partners, most of which are noncoding RNAs [29,30]. In addition to microRNAs (miRNAs), long noncoding RNAs (lncRNAs) are emerging components of EV cargo [31,32] and may be involved in the crosstalk between myeloma cells and the BM niche [33,34]. Exosomal lncRNAs contribute to the osteogenic differentiation of MM [16]. Additionally, P-element-induced wimpy testis (PIWI)-interacting RNAs (piRNAs) have been found in EVs, and EVs secreted from MM cells (MM-EVs) promote angiogenesis [17], which is one of the important processes in the development of MM [35].

The pivotal role of the BM niche in drug resistance acquisition is dependent on several factors; one of the main drivers of refractoriness are the adhesive interactions among plasma cells (PCs), BM stromal cells, and extracellular matrix (ECM) components [36]. EVs secreted from MM BM stromal cells or BM-MSCs also play essential roles in resistance to proteasome inhibitors (PIs) during the course of MM treatment [21,22].

In this review, in order thoroughly to understand key processes of MM development; osteolysis, angiogenesis, immunosuppression, ectoenzymes, and EV cargos, including several proteins and a variety of RNAs, which contribute to these processes as well as to drug resistance, are described as attractive targets for novel strategies to treat MM patients.

## 2. Ectoenzymes in the BMM

### 2.1. Expression Profiles of BMM Ectoenzymes in MM

The MM BMM includes high levels of extracellular nucleotides, such as adenosine triphosphate (ATP) and NAD^+^, which are converted to adenosine in reactions catalyzed by cell surface proteins called ectoenzymes (Figure 1) [37]. Adenosine is a nucleotide generated under metabolic stress, such as hypoxia, and acts to regulate inflammation and immune responses [38]. Extracellular ATP is hydrolyzed by the nucleoside triphosphate diphosphohydrolase CD39 to adenosine diphosphate (ADP) and then adenosine monophosphate (AMP) via the canonical adenosinergic pathway or directly via the low-affinity nucleotide pyrophosphate/phosphodiesterase (NPP) CD203a (originally identified as PC-1 [39]) (Figure 1). AMP is hydrolyzed by a 5′-nucleotidase (5′-NT), also known as NPP CD73, to generate adenosine, a potent immunosuppressor molecule. ATP is involved in the adenosinergic canonical pathway through CD39/CD73 tandem molecules on different cell surfaces (Figure 1). However, the optimal pH at which the enzyme CD39 is active is in the alkaline range of 8–8.2. The efflux of lactic acid and H^+^ induces lactic acidosis and consequently generates an acidic tumor microenvironment (TME; pH < 6.5). In contrast, the noncanonical adenosinergic pathway involves three ectoenzymes, i.e., CD38 (an NAD^+^-glycohydrolase (NADase), also known as ADP ribosyl cyclase), CD203a, and CD73 (Figure 1) [40]. In the hypoxic TME, more NAD^+^ and H^+^ are produced in the BMM, and NAD^+^ is therefore prone to metabolism via the noncanonical pathway in the MM BMM.

The distribution of ectoenzymes in the MM niche was analyzed using BM biopsies, primary PCs, and osteogenic cells from BM aspirates [11,41]. The results showed that CD203a was expressed in primary PCs and that the 5′-NT CD73 was expressed on various cells, including myeloma cells, to complete a set of noncanonical pathways to produce adenosine (Figure 1). BM biopsies [41] and BM aspirates [11] revealed that stromal cells and osteoblasts did not express either CD38 or CD39, whereas both cells expressed CD73 and CD203a. Notably, the levels of adenosine produced in the BMMs were found to be higher in the BM plasma of patients with symptomatic MM than in patients with asymptomatic MM and were correlated with disease stage according to the International Staging System (ISS) [11]. With a different substrate, e.g., ATP, AMP, or NAD^+^, an increase in adenosine production was observed when myeloma cells were cocultured with osteoclasts, osteoblasts, or BM stromal cells [11]. According to the same group, MVs isolated from the BM of patients with symptomatic MM revealed higher levels of all ectoenzymes than those from patients with MGUS/smoldering MM (SMM), and adenosine production was higher in the MVs from patients with symptomatic MM than in those from patients with MGUS/SMM [10]. These findings indicated that, in total, bulk MVs extracted from patients with MM were enriched in a mixture of ectoenzymes (CD39, CD38, CD73, and CD203a) derived from different cell components in the BM, producing adenosine by conversion of ATP (via the canonical pathway) and/or NAD^+^ (via the noncanonical pathway) in the BMM niche [10]. Because adenosine suppresses T cell proliferation and cytotoxicity, the BM niche provides an ideal location in which myeloma cells can utilize adenosine to construct a microenvironment for their survival and evasion from host immune cells.

### 2.2. Role of CD38 in Myeloma-Induced Osteoclastogenesis

CD38 is involved in rabbit [42] and mouse [43] bone resorption. Human osteoclast progenitors express CD38 on the cell surface; however, expression is lost during differentiation into mature osteoclasts in vitro, although CD14^+^ monocytes purified from peripheral blood (PB) may also be used as osteoclast progenitors [41]. Treatment with the anti-CD38 mAb daratumumab inhibits osteoclast formation in vitro, targeting early osteoclast progenitors and decreasing the area of osteoclast bone resorption [41]. These results corroborated a previous study showing that cytoplasmic CD38 expression was induced during osteoclastogenesis and that treatment with another anti-CD38 mAb, isatuximab, significantly reduced CD38 expression in osteoclasts generated from monocytes cultured with the osteoclast-activating factor receptor activator of nuclear factor-κB ligand (RANKL) and macrophage colony-stimulating factor ex vivo without affecting osteoclast formation. Furthermore, restoration of the T cell response by isatuximab was also attributed to the downregulation of the expression of herpesvirus entry mediator (HVEM) and indoleamine-2,3-dioxygenase (IDO) [44].

### 2.3. Role of CD38 in Bioenergetic Plasticity in MM through Mitochondrial Transfer

The levels of glycolysis are lower in primary myeloma cells than in myeloma cell lines. This observation led to the finding that myeloma cells use mitochondrion-based metabolism as well as glycolysis when they are grown in direct contact with primary BM stromal cells. The mitochondrial metabolic state of BM stromal cells plays a role in the bioenergetic flexibility of myeloma cells cocultured with BM stromal cells. Myeloma cells obtain mitochondria from BM stromal cells, favoring oxidative phosphorylation via tumor-derived tunneling nanotubes. Moreover, a CD38-blocking antibody was found to significantly reduce mitochondrial transfer and mitochondrial oxidative metabolism in myeloma cells cocultured with BM stromal cells [45].

## 3. The Immunosuppressive Role of Osteoclasts in the BMM

**Figure 2:**
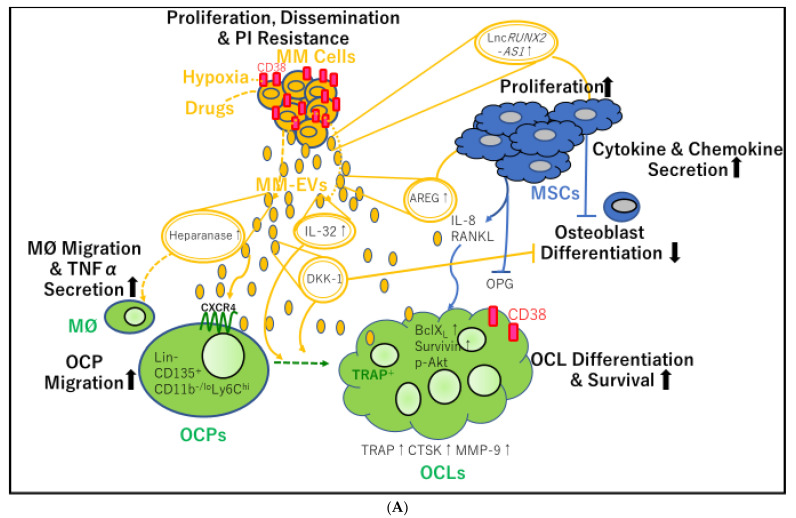

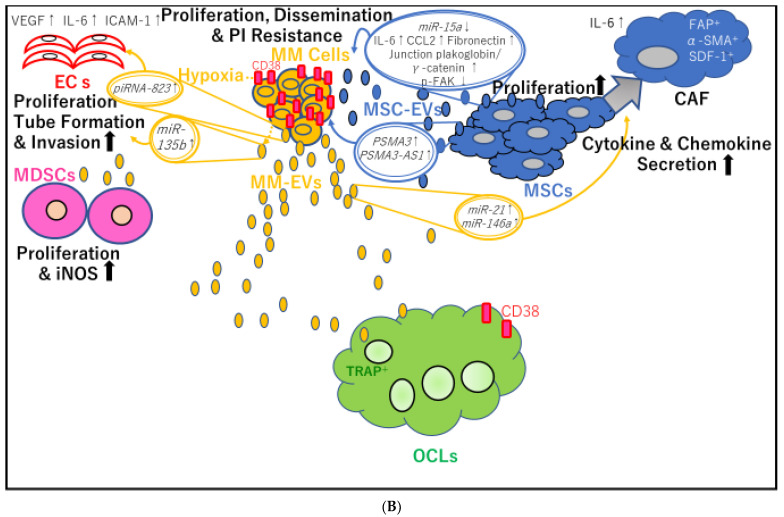
Mutual transfer of different molecules contained in extracellular vesicle (EV) cargo between multiple myeloma cell-derived EVs (MM-EVs) and bone marrow mesenchymal stem cell (BM-MSC)-derived EVs (MSC-EVs). (**A**) MM-EVs contribute to osteoclast (OCL) precursor (OCP) migration, survival, differentiation, and osteolysis. MM-EVs also inhibit osteoblast differentiation, either directly or by stimulating BM-MSCs. PI: proteasome inhibitor; MM: multiple myeloma; lnc: long noncoding; AS: antisense; MM-EVs: multiple myeloma cell-derived extracellular vesicles; IL: interleukin; AREG: amphiregulin; MSCs: mesenchymal stem cells; RANKL: receptor activator of nuclear factor-κB ligand; MØ: macrophage; TNFα: tumor necrosis factor-α; OCP: osteoclast precursors; CXCR4: C-X-C chemokine receptor type 4; DKK-1: Dickkopf-1; OPG: osteoprotegerin; TRAP: tartrate-resistant acid phosphatase; BCLX_L_: B-cell lymphoma-extra large; p-AKT: phosphorylated protein kinase B; CTSK: cathepsin K; MMP-9: matrix metalloprotease-9; OCL: osteoclast. (**B**) Interaction between myeloma cells and their microenvironment through EVs containing PIWI-interacting RNAs (piRNAs), long noncoding RNAs (lncRNAs), and proteins, contributing to angiogenesis, MDSC proliferation, and cancer-associated fibroblast (CAF) transformation of MSCs. VEGF: vascular endothelial growth factor; IL: ICAM-1: interleukin; intercellular adhesion molecule-1; ECs: endothelial cells; piRNA: P-Element induced wimpy testis (PIWI)-interacting RNA; miR: microRNA; MM: multiple myeloma; CCL2: C-C motif chemokine 2; p-FAK, phospho-focal adhesion kinase; CAF: cancer associated fibroblast; MSC-EVs: mesenchymal stromal cell-derived extracellular vesicles; PSMA3: proteasome 20S subunit alpha 3; MSCs: mesenchymal stem cells; MDSCs; myeloid-derived suppressor cells; MM-EVs: multiple myeloma-derived extracellular vesicles; iNOS: inducible nitric oxidase synthase; TRAP: tartrate-resistant acid phosphatase; OCL: osteoclast.

Osteoclasts induce the expression of FoxP3 on activated CD44^+^CD8^+^ T cells and suppress the priming of naïve CD8^+^ T cells [46]. Primary murine BM osteoclast precursors belong to a CD11b^−/low^Ly6C^high^ population and do not express Ly6G [47] (Figure 2A). With reference to MDSCs, monocytic MDSCs (M-MDSCs) express CD11b and Ly6C, whereas granulocytic myeloid-derived suppressor cells (G-MDSCs) express CD11b and Ly6G [48]. Analogous to M-MDSCs, osteoclast precursors suppress CD8^+^ and CD4^+^ T cell proliferation; the latter is mediated by the production of nitric oxide [47].

Osteoclasts protect myeloma cells against T cell-mediated anti-MM immunity. The potential of osteoclasts to promote immunosuppressive effects on T cells is attributed to the elevation of several co-inhibitory molecules, such as PD-L1, galectin-9, HVEM, and CD200. Moreover, osteoclasts from patients with MM strongly generate IDO, the levels of which are significantly higher in the BM plasma of patients with MM than in those of healthy donors (HDs). The expression levels of these molecules are higher in patients with newly diagnosed MM than in HDs [44].

Notably, a proliferation-inducing ligand (APRIL) accelerates myeloma cell survival and disease progression in vivo [49]. Osteoclasts are the major source of APRIL in the BMM, and PD-L1 expression in MM cells is modulated by the APRIL/B-cell maturation antigen signaling cascade through the mitogen-activated protein kinase (MAPK)/extracellular signal-regulated kinase (ERK) pathway [49].

## 4. EVs

### 4.1. BM-MSC-Derived Exosomes Support Myeloma Cell Dissemination

As listed in Table 1, MM BM-MSCs secrete exosomes (MM BM-MSC-exos) with an altered composition compared with exosomes produced by HD BM-MSCs (HD BM-MSC-exos). MM BM-MSC-exos promote the growth and dissemination of myeloma cells in SCID-beige mice, in contrast to HD BM-MSC-exos [20]. Additionally, a higher tumor growth rate was observed in recipients of tissue-engineered bone implants loaded with myeloma cells exposed to MM BM-MSC-exos than in those receiving bone implants loaded with only myeloma cells [20]. MicroRNA (miR) expression profiling revealed that *miR-15a* was dramatically downregulated in MM BM-MSC-exos and that the levels of the tumor suppressor *miR-15a* in BM-MSC-exos from patients with MGUS were higher than in those from patients with MM. Primary HD BM-MSCs express higher levels of *miR-15a* than primary MM BM-MSCs, and *miR-15a* is significantly upregulated in MM cells when cocultured with HD BM-MSCs, but not when cocultured with MM BM-MSCs. Thus, *miR-15a* is transferred from HD BM-MSCs to MM PCs, and normal BM-MSC-exos inhibit myeloma cell proliferation. In addition, proteomic analysis of MM BM-MSC-exos showed elevated levels of oncogenic proteins (such as junction plakoglobin (also known as γ catenin)), cytokines/chemokines (such as interleukin (IL)-6 and C-C motif chemokine ligand (CCL2)), and adhesion molecules (including fibronectin), all of which promote the dissemination of MM cells to the distant BM niche in contrast to HD BM-MSC-exos [20] (Figure 2B).

### 4.2. BM Stromal Cell-Derived EVs Confer Resistance to PIs in Myeloma Cells

Using the 5T33MM mouse model, a previous study reported that 5T33 BM stromal cell-derived exosomes promoted the survival and proliferation of 5T33MMvt and 5T33MMvv cells; however, the same effects were observed in HD BM stromal cells [21]. This finding indicated that the secretion of BM stromal cells may favor myeloma cell migration, growth, and survival, regardless of the disease or normal BMM status. Moreover, BM stromal cell-derived exosomes induce resistance of RPMI8226 myeloma cells to bortezomib, which activates several survival-relevant pathways, including c-Jun N-terminal kinase (JNK), p38, p53, and protein kinase B (AKT) [21].

### 4.3. MSC-Derived Exosomes Confer Resistance to PIs in Myeloma Cells

In MSCs, *PSMA3*, which encodes proteasome 20S subunit α3 and lnc*PSMA3* antisense mRNA (lnc*PSMA3*-antisense (AS)1)—which arises from the antisense strand of *PSMA3*—can be packed into exosomes and transferred to myeloma cells to render cells resistance to PIs in myeloma cells [22]. *PSMA3-AS1* assembles an RNA duplex with pre-*PSMA3*, which transcriptionally promotes *PSMA3* expression by enhancing its stability via suppression of decay. The expression of *PSMA3* mRNA is higher in CD138^+^ cells from patients with MM than in those from patients with MGUS, and the same is true for CD138^+^ cells from patients with PC leukemia compared with those from patients with MM. Thus, *PSMA3* expression in CD138^+^ cells may be associated with disease progression. Pearson correlation analysis revealed a positive correlation between the gene expression of *PSMA3* or *PSMA3-AS1* in CD138^+^ myeloma cells and that in circulating exosomes secreted from patients with MM. Moreover, circulating exosomal *PSMA3* and *PSMA3-AS1* in plasma from patients with MM are significantly correlated with progression-free survival (PFS) and OS. In addition, in xenograft models, an intravenously administered small interfering RNA targeting *PSMA3-AS1* efficiently increases sensitivity to another PI, carfilzomib [22] (Figure 2B).

In addition to exosomes, myeloma cells cultured with MM BM-MSC MVs exhibit rapid (5 min) and sustained (24 h) activation of MAPK and eukaryotic translation initiation (TI) factor 4 following MV uptake by myeloma cells [50]. According to further analysis from the same group, compared with HD BM-MSC decellularized ECM, myeloma cells cultured on MM BM-MSC ECM show activated MAPK/TI, proliferation, migration, invasion, epithelial-to-mesenchymal transition (EMT), and C-X-C chemokine receptor 4 (CXCR4) expression in myeloma cells [51]. A previous report by Roccaro et al. showed that CXCR4 regulates EMT in MM and that PCs overexpressing CXCR4 are more prone to bone dissemination [52]. Furthermore, myeloma cells exposed to MM BM-MSC ECM exhibit an increase in autophagy levels to a greater extent than those exposed to HD BM-MSC ECM. Secreted MM BM-MSC MVs can bind to the ECM, internalize ECM-associated myeloma cells, and increase the resistance of myeloma cells to doxorubicin and bortezomib [51]. However, to identify novel therapeutic targets, it is necessary to further characterize the differences in ECM components and MV cargo between HD BM-MSCs and MM BM-MSCs.

### 4.4. MM-EVs Promote the Formation of New Bone Lesions

Osteoclasts in MM are derived from monocytes [53] and MDSCs [54]. Using murine peritoneal RAW264.7 macrophages as osteoclast precursors, treatment with human recombinant RANKL confirmed the presence of tartrate-resistant acid phosphatase (TRAP)-positive multinucleated osteoclasts [55]. In these experiments, the number of TRAP-positive multinucleated osteoclasts increased significantly in the presence of MM cell-derived exosomes (MM-exos). Moreover, MM-exos contributed to osteoclast migration by increasing CXCR4 expression in vitro and promoted the modulation of osteoclast differentiation by increasing the levels of the anti-apoptotic proteins B cell lymphoma-extra-large (Bcl-X_L_) (also known as BCL-2-like 1), survivin, and phosphorylated AKT (Figure 2A). As a result, MM-exos induced RAW264.7-derived osteoclasts, which exhibited authentic bone resorption lacunae in the dentine substrate. MM-exos regulated osteoclast bone reabsorption by increasing the expression of key osteoclastogenic enzymes, such as TRAP, cathepsin K, and matrix metalloproteinase-9 [55] (Figure 2A).

The functional imbalance between osteoclasts and osteoblasts is correlated with increased osteoclast lytic activity during MM progression [56]. Zhang et al. reported a correlation between CD138^+^ circulating EVs in PB and bone lesions in patients with MM. In addition, they revealed that MM-EVs significantly elevated the expression of *miR-103a-3p* in BM-MSCs, which exhibited inhibitory effects on the osteogenic differentiation of BM-MSCs. Furthermore, they showed that MM-EVs injected into the tibia in mice led to impaired osteogenesis and exacerbated MM bone diseases. Collectively, these findings suggested that MM-EVs may play a role in promoting bone disease spreading in MM [56].

Recent studies have shown that several protein cargoes of MM-exos contribute to bone resorption (Figure 2A). First, the pro-inflammatory cytokine IL-32 was identified as a protein delivered via MM-EVs and was shown to play key roles in promoting osteoclast activity [14]. At diagnosis, patients with MM who expressed *IL32* were found to have more advanced disease than patients who did not express *IL-32*. Furthermore, PFS was shown to be significantly shorter in patients with higher *IL-32* expression than in those with lower *IL-32* expression. Both *IL-32* mRNA and IL-32 protein were found to be increased in response to hypoxia in myeloma cells, and protein levels of IL-32 were shown to be dependent on the expression of hypoxia-inducible factor (HIF)-1α. Additionally, gene set analyses showed that high *IL-32* expression was significantly associated with a hypoxic signaling pathway. MM-EVs from wild-type high IL-32-expressing JJN3 myeloma cells were found to promote osteoclast differentiation, whereas pro-osteoclasts treated with EVs from *IL-32*-knockout (KO) JJN3 cells did not. Furthermore, micro-computed tomography images showed extensive osteolytic lesions in the tibiae of mice injected with wild-type JJN3 cells compared with those of mice injected with *IL-32*-KO JJN3 cells. Finally, patients with focal bone lesions assessed by magnetic resonance imaging were found to have significantly higher *IL-32* expression in myeloma cells than patients without bone lesions, suggesting that IL-32 may play a role in MM bone diseases [14]. Interestingly, a previous report showed that HIF-1α knockdown in cells overexpressing IL-32 reduced macrophage inflammatory protein-1α (MIP1-α)/CCL3 levels and caused nearly complete bone destruction in mice [57].

Notably, treatment with bortezomib, carfilzomib, or melphalan increased the levels of heparanase present in exosomes, which induced macrophage migration and TNFα secretion [23] and may eventually promote osteoclast differentiation. Additionally, 5TGM1-derived small EVs (probably exosomes, referred to as 5TGM1-exos) containing the osteogenic Wnt pathway inhibitor Dickkopf-1 (DKK-1) were found to induce apoptosis, upregulate *DKK-1*, and reduce runt-related transcription factor 2 (RUNX2) levels in undifferentiated osteoblastic cells. Moreover, when 5TGM1-exos were added to the culture medium of differentiated osteoblasts, the expression of *ALP* and *collagen 1A1* genes, both of which encode proteins produced by terminally differentiated osteoblasts, was diminished. In contrast, a significant reduction in the trabecular bone of femurs was induced in mice injected with 5TGM1-exos. Consequently, the sphingomyelinase inhibitor GW4869, which inhibits the secretion of exosomes, caused an increase in cortical bone volume when combined with bortezomib treatment [15].

Finally, the epidermal growth factor receptor (EGFR) ligand amphiregulin (AREG) was identified as another protein delivered by MM-exos and was shown to be involved in osteoclastogenesis. In murine RAW264.7 cells and human PB CD14^+^ cells as osteoclast progenitor cells, MM-exos were found to reduce mineralized nodules and *OPG* expression but they increased *RANKL* expression, EGFR activation, and IL-8 release in human MSC cells. Accordingly, pretreatment with anti-AREG mAbs was found to abrogate EGFR activation and increase IL-8 levels [24]. Thus, via enhanced osteoclast activity and reduced osteoblast differentiation, MM-exos loaded with DKK-1 and AREG may support the formation of bone lytic lesions (Table 1).

RUNX2 can also be suppressed by MM-exos through transfer of the lncRNA *RUNX2-AS1* in MSCs. *RUNX2-AS1*, which arises from the antisense strand of *RUNX2*, is enriched in MM-MSCs and forms an RNA duplex with *RUNX2* premRNA through its overlapping sequences. The duplex reduces splicing efficiency, transcriptionally represses *RUNX2* expression, and decreases MSC osteogenic potential. Moreover, in NOD-Prkdc scid Il2rg^−/−^ mice, administration of GW4869 increases bone formation and alters bone turnover markers. Accordingly, the exosomal lncRNA *RUNX2-AS1* is a potential therapeutic target for bone lesions in MM [16].

### 4.5. MM-EVs Promote Angiogenesis

A previous study showed that under normoxic or acute hypoxic conditions, the hypoxia-resistant MM cells RPMI8226-HR produce more exosomes than the parental cells and that *miR-135b* is significantly increased in exosomes from RPMI8226-HR cells. Exosomal *miR-135b* enhances endothelial tube formation (Figure 2B) under hypoxia through HIF-1, thereby inhibiting signaling pathways involving HIF-1, which has an *miR-135* binding site in its 3′-untranslated region [18]

*piRNA-823* [17] contributes to the angiogenic effects of MM-EVs. piRNAs belong to a class of small noncoding RNAs, 24–32 nucleotides in length. piRNAs bind to PIWI proteins to assemble into the piRNA/PIWI-complex, which epigenetically and post-transcriptionally silences transposable elements in germline stem cells [58,59]. Moreover, piRNAs and PIWI proteins are aberrantly expressed in various cancers, and piRNAs/PIWI complexes are involved in tumor progression [60] and tumorigenesis [61]. In MM, three reports have described roles of *piRNA-823*. First, this piRNA has been shown to be implicated in tumorigenesis by modulating DNA methylation of the gene promoter region of putative tumor-suppressor genes, such as *p16^INK4A^*, and by regulating angiogenesis. Interestingly, *piRNA-823* expression is increased in myeloma cell lines and patients with primary MM according to the ISS [62]. Because *piRNA-823* is directly related to DNA methyltransferase 3A (DNMT3A) and 3B (DNMT3B) in myeloma cells, *piRNA-823* inhibition using a *piRNA-823* antagomir (antagomir-823) was shown to result in significant decreases in DNMT3A and DNMT3B expression. In addition, vascular endothelial growth factor (VEGF) production is significantly reduced in conditioned medium from myeloma cells transfected with antagomir-823. Accordingly, coculture of ECs with antagomir-823-transfected myeloma cells attenuates the activation of ERK and AKT pathways, decreases chemotactic motility, and results in incomplete tube structure formation in ECs [62]. Furthermore, *piRNA-823* elevates IL-6 and VEGF secretion in cultured ECs and promotes the expression of intercellular adhesion molecule-1 and CXCR4 in ECs. Consequently, transfection with a *piRNA-823* mimic facilitates the proliferation, fusion, and invasion of ECs [17] (Figure 2B). In addition, *piRNA-823* suppresses apoptosis in ECs following the efficient transfer of MM-EVs to ECs [17]. G-MDSCs have also been shown to enhance the stemness of MM stem cells via upregulation of *piRNA-823* expression. Myeloma cells cocultured with G-MDSCs induce DNMT3B expression, and antagomir-823 transfection reverses this effect in myeloma cells. Thus, *piRNA-823* modulates MM stemness by activating DNMT3B [63].

By contrast, a previous study showed that murine MM-exos harbor multiple angiogenesis-related proteins, including angiogenin and VEGF [19] (Table 1)). In ECs and BM stromal cells, 5T3MMvt exosomes modulate STAT3, JNK, and p53, thereby enhancing the viability of BM ECs and promoting BM stromal cell growth [19].

### 4.6. MM-EVs Induce Immunosuppression in the BMM in MM

Immunosuppressive MDSCs accumulate in the BMM in the early stages of tumor development [64] and are a prominent immune population mediating MM progression by suppressing T cell activation [65] and inducing MM cell survival [66]. Intriguingly, 5T33MMvt cell-derived EVs promote the viability and proliferation of MDSCs by activating the signal transducer and activator of transcription 3 (STAT3) pathway, upregulating Bcl-X_L_ and myeloid cell leukemia-1, and promoting inducible nitric oxide synthase expression [19] (Figure 2B).

### 4.7. MM-Exos Transform MSCs into Cancer-Associated Fibroblasts (CAFs)

Carrying high levels of *miR-21* and *miR-146a*, exosomes derived from OPM-2 MM cells were found to promote MSC proliferation via increased *miR-21* and *miR-146a* expression in MSCs. Furthermore, CAF transformation was induced as a result of increased mRNA expression of CAF markers, including fibroblast-activating protein, α-smooth-muscle actin, and stromal-derived factor 1, as well as enhanced IL-6 secretion [25] (Figure 2B).

## 5. Conclusions

In summary, by targeting myeloma cells and reducing the levels of immunosuppressive adenosine, the anti-CD38 antibody daratumumab is expected to improve the quality of remission in patients with MM. In particular, in the hypoxic state in the BM niche, isatuximab has a stronger ability to inhibit CD38-NADase activity than daratumumab [8]. By elucidating the contents of exosomes, new approaches using exosomes purified from immune cells other than MSCs may be developed in the future. In particular, strategies to reverse PI resistance are needed to facilitate the delivery to the BM.

## Figures and Tables

**Table 1:** Alterations in the exosomal content of MM patients compared to healthy donors.

Ex or EV Origin	Content	Mode of Alteration	Function	Ref.
BM-MSCs	Oncosuppressor *miR-15a*	↓	MM cell proliferation and dissemination	[20]
	IL-6, CCL2, fibronectin, junction plakoglobin/γ-catenin	↑		
	p-FAK	↓		
BMSCs (murine 5T33 MM model)	unknown	↑	MM cell viability, proliferation, survival, and migration and bortezomib resistance	[21]
BM-MSCs	*PSMA3, PSMA3-AS1*	↑	PI resistance	[22]
RPMI8226, OPM-2, LP-1, U266	*miR-146a*	↑	Increase cytokine and chemokine secretion from MSCs	[13]
CAG, RPMI8226	heparanase	↑ (bortezomib, carfilzomib, melphalan treatment)	Stimulate macrophage migration and secretion of TNF-α	[23]
JJN3, H929, primary MM cells	IL-32	↑	Promote OCL differentiation	[14]
MM.1S	AREG (EGFR ligand)	↑	Osteolysis by promoting OCL differentiation and blocking osteogenic differentiation	[24]
Murine MM cell 5TGM1	DKK-1	-	Osteolysis by promoting OCL differentiation and blocking osteoblast differentiation	[15]
MM cells	*lncRNA RUNX2-AS1*	↑	Reduce osteogenic differentiation	[16]
RPMI8226, U266m ARH-77, primary MM cells	*piRNA-823*	↑	Promote proliferation, tube formationm and invasion of ECs	[17]
RPMI8226-HR	*miR-135b*	↑	Promote tube formation of ECs	[18]
Murine MM cell 5T3MMvt, RPMI8226	Angiogenin, VEGF, seepine1/PAI1, TIMP-1,	-	Promote angiogenesis, MDSC viability, and proliferation	[19]
OPM-2	*miR-21* & *miR-146a*	↑	MSC proliferation, CAF transformation, and IL-6 secretion	[25]

Ex, exosome; EV, extracellular vesicles; BM, bone marrow; MSC, mesenchymal stem cell; miR, microRNA; ↓, down-regulated; IL, interleukin; CCL2, C-C motif chemokine 2; ↑, up-regulated; p-FAK, phospho-focal adhesion kinase; BMSCs, bone marrow stromal cells; MM, multiple myeloma; *PSMA3,* proteasome 20S subunit α3; PI, proteasome inhibitor; TNFα, tumor necrotizing factor α; OCL, osteoclast; AREG, amphiregulin; EGFR, epithelial growth factor receptor; DKK-1, Dickkopf-1; lncRNA, long non-coding RNA; RUNX2, runt-related transcription factor 2; AS, antisense; piRNA, P-Element induced wimpy testis (PIWI)-interacting RNA; ECs, endothelial cells; RPMI8226-HR, hypoxia-resistant RPMI8226; VEGF, vascular endothelial growth factor; PAI1: plasminogen activator inhibitor-1; TIMP, tissue inhibitor of metalloprotease; MDSC, myeloid-derived suppressor cell; CAF, cancer-associated fibroblast.

## References

[1] Anderson K.C., Carrasco R.D. (2011). Pathogenesis of Myeloma. Annu. Rev. Pathol..

[2] Kyle R.A., Therneau T.M., Rajkumar S.V., Offord J.R., Larson D.R., Plevak M.F., Melton L.J. (2002). A long-term study of prognosis in monoclonal gammopathy of undetermined significance. N. Engl. J. Med..

[3] Kyle R.A., Vincent Rajkumar S.V. (2006). Monoclonal gammopathy of undetermined significance. Br. J. Haematol..

[4] Kumar S.K., Dispenzieri A., Lacy M.Q., Gertz M.A., Buadi F.K., Pandey S., Kapoor P., Dingli D., Hayman S.R., Leung N. (2014). Continued improvement in survival in multiple myeloma: Changes in early mortality and outcomes in older patients. Leukemia.

[5] Robak P., Drozdz I., Szemraj J., Robak T. (2018). Drug resistance in multiple myeloma. Cancer Treat. Rev..

[6] Facon T., Kumar S., Plesner T., Orlowski R.Z., Moreau P., Bahlis N., Basu S., Nahi H., Hulin C., Quach H. (2019). Daratumumab plus Lenalidomide and Dexamethasone for Untreated Myeloma. N. Engl. J. Med..

[7] Mateos M.-V., Cavo M., Blade J., Dimopoulos M.A., Suzuki K., Jakubowiak A., Knop S., Doyen C., Lucio P., Nagy Z. (2020). Overall survival with daratumumab, bortezomib, melphalan, and prednisone in newly diagnosed multiple myeloma (ALCYONE): A randomised, open-label, phase 3 trial. Lancet.

[8] Martin T.G., Corzo K., Chiron M., Van De Velde H., Abbadessa G., Campana F., Solanki M., Meng R., Lee H., Wiederschain D. (2019). Therapeutic Opportunities with Pharmacological Inhibition of CD38 with Isatuximab. Cells.

[9] Morandi F., Morandi B., Horenstein A.L., Chillemi A., Quarona V., Zaccarello G., Carrega P., Ferlazzo G., Mingari M.C., Moretta L. (2015). A non-canonical adenosinergic pathway led by CD38 in human melanoma cells induces suppression of T cell proliferation. Oncotarget.

[10] Morandi F., Marimpietri D., Horenstein A.L., Bolzoni M., Toscani D., Costa F., Castella B., Faini A.C., Massaia M., Pistoia V. (2018). Microvesicles released from multiple myeloma cells are equipped with ectoenzymes belonging to canonical and non-canonical adenosinergic pathways and produce adenosine from ATP and NAD+. OncoImmunology.

[11] Horenstein A.L., Quarona V., Toscani D., Costa F., Chillemi A., Pistoia V., Giuliani N., Malavasi F. (2016). Adenosine Generated in the Bone Marrow Niche through a CD38-Mediated Pathway Correlates with Progression of Human Myeloma. Mol. Med..

[12] Horenstein A.L., Bracci C., Morandi F., Malavasi F. (2019). CD38 in Adenosinergic Pathways and Metabolic Re-programming in Human Multiple Myeloma Cells: In-tandem Insights from Basic Science to Therapy. Front. Immunol..

[13] De Veirman K., Wang J., Xu S., Leleu X., Himpe E., Maes K., De Bruyne E., Van Valckenborgh E., Vanderkerken K., Menu E. (2016). Induction of miR-146a by multiple myeloma cells in mesenchymal stromal cells stimulates their pro-tumoral activity. Cancer Lett..

[14] Zahoor M., Westhrin M., Aass K.R., Moen S.H., Misund K., Psonka-Antonczyk K.M., Giliberto M., Buene G., Sundan A., Waage A. (2017). Hypoxia promotes IL-32 expression in myeloma cells, and high expression is associated with poor survival and bone loss. Blood Adv..

[15] Faict S., Muller J., De Veirman K., De Bruyne E., Maes K., Vrancken L., Heusschen R., De Raeve H., Schots R., Vanderkerken K. (2018). Exosomes play a role in multiple myeloma bone disease and tumor development by targeting osteoclasts and osteoblasts. Blood Cancer J..

[16] Li B., Xu H., Han H., Song S., Zhang X., Ouyang L., Qian C., Hong Y., Qiu Y., Zhou W. (2018). Exosome-mediated transfer of lncRUNX2-AS1 from multiple myeloma cells to MSCs contributes to osteogenesis. Oncogene.

[17] Li B., Hong J., Hong M., Wang Y., Yu T., Zang S., Wu Q. (2019). piRNA-823 delivered by multiple myeloma-derived extracellular vesicles promoted tumorigenesis through re-educating endothelial cells in the tumor environment. Oncogene.

[18] Umezu T., Tadokoro H., Azuma K., Yoshizawa S., Ohyashiki K., Ohyashiki J.H. (2014). Exosomal miR-135b shed from hypoxic multiple myeloma cells enhances angiogenesis by targeting factor-inhibiting HIF-1. Blood.

[19] Wang J., De Veirman K., Faict S., Frassanito M.A., Ribatti D., Vacca A., Menu E. (2016). Multiple myeloma exosomes establish a favourable bone marrow microenvironment with enhanced angiogenesis and immunosuppression. J. Pathol..

[20] Roccaro A.M., Sacco A., Maiso P., Azab A.K., Tai Y.T., Reagan M., Azab F., Flores L.M., Campigotto F., Weller E. (2013). BM mesenchymal stromal cell-derived exosomes facilitate multiple myeloma progression. J. Clin. Investig..

[21] Wang J., Hendrix A., Hernot S., Lemaire M., De Bruyne E., Van Valckenborgh E., Lahoutte T., De Wever O., Vanderkerken K., Menu E. (2014). Bone marrow stromal cell–derived exosomes as communicators in drug resistance in multiple myeloma cells. Blood.

[22] Xu H., Han H., Song S., Yi N., Qian C., Qiu Y., Zhou W., Hong Y., Zhuang W., Li Z. (2019). Exosome-Transmitted PSMA3 and PSMA3-AS1 Promote Proteasome Inhibitor Resistance in Multiple Myeloma. Clin. Cancer Res..

[23] Bandari S.K., Purushothaman A., Ramani V.C., Brinkley G., Chandrashekar D.S., Varambally S., Mobley J.A., Zhang Y., Brown E.E., Vlodavsky I. (2018). Chemotherapy induces secretion of exosomes loaded with heparanase that degrades extracellular matrix and impacts tumor and host cell behavior. Matrix Biol..

[24] Raimondo S., Saieva L., Vicario E., Pucci M., Toscani D., Manno M., Raccosta S., Giuliani N., Alessandro R. (2019). Multiple myelo-ma-derived exosomes are enriched of amphiregulin (AREG) and activate the epidermal growth factor pathway in the bone microenvironment leading to osteoclastogenesis. J. Hematol. Oncol..

[25] Cheng Q., Li X., Liu J., Ye Q., Chen Y., Tan S., Liu J. (2017). Multiple Myeloma-Derived Exosomes Regulate the Functions of Mesenchymal Stem Cells Partially via Modulating miR-21 and miR-146a. Stem Cells Int..

[26] Zhang H., Freitas D., Kim H.S., Fabijanic K., Li Z., Chen H., Mark M.T., Molina H., Martin A.B., Bojmar L. (2018). Identification of distinct nanoparticles and subsets of extracellular vesicles by asymmetric flow field-flow fractionation. Nat. Cell Biol..

[27] Verdera H.C., Gitz-Francois J.J., Schiffelers R.M., Vader P. (2017). Cellular uptake of extracellular vesicles is mediated by clath-rin-independent endocytosis and macropinocytosis. J Controll Release.

[28] Mathieu M., Martin-Jaular L., Lavieu G., Théry C. (2019). Specificities of secretion and uptake of exosomes and other extracellular vesicles for cell-to-cell communication. Nat. Cell Biol..

[29] He Y., Vogelstein B., Velculescu V., Papadopoulos N., Kinzler K.W. (2008). The Antisense Transcriptomes of Human Cells. Science.

[30] Wang P., Xue Y., Han Y., Lin L., Wu C., Xu S., Jiang Z., Xu J., Liu Q., Cao X. (2014). The STAT3-Binding Long Noncoding RNA lnc-DC Controls Human Dendritic Cell Differentiation. Science.

[31] Qu L., Ding J., Chen C., Wu Z., Liu B., Gao Y., Chen W., Liu F., Sun W., Li X.-F. (2016). Exosome-transmitted lncARSR promotes sunitinib resistance in renal cancer by acting as a competing endogenous RNA. Cancer Cell.

[32] Mohankumar S., Patel T. (2016). Extracellular vesicle long noncoding RNA as potential biomarkers of liver cancer. Briefings Funct. Genom..

[33] Amodio N., Raimondi L., Juli G., Stamato M.A., Caracciolo D., Tagliaferri P., Tassone P. (2018). MALAT1: A druggable long non-coding RNA for targeted anti-cancer approaches. J. Hematol. Oncol..

[34] Butova R., Vychytilova-Faltejskova P., Souckova A., Sevcikova S., Hajek R. (2019). Long Non-Coding RNAs in Multiple Myeloma. Non-Coding RNA.

[35] Ribatti D., Vacca A. (2018). New Insights in Anti-Angiogenesis in Multiple Myeloma. Int. J. Mol. Sci..

[36] Di Marzo L., Desantis V., Solimando A.G., Ruggieri S., Annese T., Nico B., Fumarulo R., Vacca A., Frassanito M.A. (2016). Microenvironment drug resistance in multiple myeloma: Emerging new players. Oncotarget.

[37] Horenstein A.L., Chillemi A., Quarona V., Zito A., Roato I., Morandi F., Marimpietri D., Bolzoni M., Toscani D., Oldham R.J. (2015). NAD+-Metabolizing Ectoenzymes in Remodeling Tumor–Host Interactions: The Human Myeloma Model. Cells.

[38] Antonioli L., Blandizzi C., Pacher P., Haskó G. (2013). Immunity, inflammation and cancer: A leading role for adenosine. Nat. Rev. Cancer.

[39] Takahasi T., Old L.J., Boyse E.A. (1970). Surface antigens of immunocompetent cells. I. Effect of theta and PC.1 alloantisera on the ability of spleen cells to transfer immune responses. J. Exp. Med..

[40] Horenstein A.L., Chillemi A., Zaccarello G., Bruzzone S., Quarona V., Zito A., Serra S., Malavasi F. (2013). A CD38/CD203a/CD73 ecto-enzymatic pathway independent of CD39 drives a novel adenosinergic loop in human T lymphocytes. Oncoimmunology.

[41] Costa F., Toscani D., Chillemi A., Quarona V., Bolzoni M., Marchica V., Vescovini R., Mancini C., Martella E., Campanini N. (2017). Expression of CD38 in myeloma bone niche: A rational basis for the use of anti-CD38 immunotherapy to inhibit osteoclast formation. Oncotarget.

[42] Sun L., Adebanjo O.A., Moonga B.S., Corisdeo S., Anandatheerthavarada H.K., Biswas G., Arakawa T., Hakeda Y., Koval A., Sodam B. (1999). CD38/ADP-ribosyl cyclase: A new role in the regulation of osteoclastic bone resorption. J. Cell Biol..

[43] Sun L., Iqbal J., Dolgilevich S., Yuen T., Wu X.-B., Moonga B.S., Adebanjo O.A., Bevis P.J.R., Lund F., Huang C.L.-H. (2003). Disordered osteoclast formation and function in a CD38 (ADP-ribosyl cyclase)-deficient mouse establishes an essential role for CD38 in bone resorption. FASEB J..

[44] An G., Acharya C., Feng X., Wen K., Zhong M., Zhang L., Munshi N.C., Qiu L., Tai Y.-T., Anderson K.C. (2016). Osteoclasts promote immune suppressive microenvironment in multiple myeloma: Therapeutic implication. Blood.

[45] Marlein C.R., Piddock R.E., Mistry J.J., Zaitseva L., Hellmich C., Horton R.H., Zhou Z., Auger M.J., Bowles K.M., Rushworth S.A. (2019). CD38-Driven Mitochondrial Trafficking Promotes Bioenergetic Plasticity in Multiple Myeloma. Cancer Res..

[46] Kiesel J.R., Buchwald Z.S., Aurora R. (2009). Cross-presentation by osteoclasts induces FoxP3 in CD8^+^ T cells. J. Immunol..

[47] Charles J.F., Hsu L.Y., Niemi E.C., Weiss A., Aliprantis A.O., Nakamura M.C. (2012). Inflammatory arthritis increases mouse osteoclast precursors with myeloid suppressor function. J. Clin. Investig..

[48] Marvel D., Gabrilovich D.I. (2015). Myeloid-derived suppressor cells in the tumor microenvironment: Expect the unexpected. J. Clin. Investig..

[49] Tai Y.-T., Acharya C., An G., Moschetta M., Zhong M.Y., Feng X., Cea M., Cagnetta A., Wen K., van Eenennaam H. (2016). APRIL and BCMA promote human multiple myeloma growth and immunosuppression in the bone marrow microenvironment. Blood.

[50] Dabbah M., Attar-Schneider O., Matalon S.T., Shefler I., Dolberg O.J., Lishner M., Drucker L. (2017). Microvesicles derived from normal and multiple myeloma bone marrow mesenchymal stem cells differentially modulate myeloma cells’ phenotype and translation initiation. Carcinogenesis.

[51] Ibraheem A., Attar-Schneider O., Dabbah M., Jarchowsky O.D., Matalon S.T., Lishner M., Drucker L. (2019). BM-MSCs-derived ECM modifies multiple myeloma phenotype and drug response in a source-dependent manner. Transl. Res..

[52] Roccaro A.M., Mishima Y., Sacco A., Moschetta M., Tai Y.T., Shi J., Zhang Y., Reagan M.R., Huynh D., Kawano Y. (2015). CXCR4 regulates extra-medullary myeloma through epi-thelial-mesenchymal-transition-like transcriptional activation. Cell Rep..

[53] Massey H.M., Flanagan A.M. (1999). Human osteoclasts derive from CD14-positive monocytes. Br. J. Haematol..

[54] Zhuang J., Zhang J., Lwin S.T., Edwards J.R., Edwards C.M., Mundy G.R., Yang X. (2012). Osteoclasts in Multiple Myeloma Are Derived from Gr-1+CD11b+Myeloid-Derived Suppressor Cells. PLoS ONE.

[55] Raimondi L., De Luca A., Amodio N., Manno M., Raccosta S., Taverna S., Bellavia D., Naselli F., Fontana S., Schillaci O. (2015). In-volvement of multiple myeloma cell-derived exosomes in osteoclast differentiation. Oncotarget.

[56] Zhang L., Lei Q., Wang H., Xu C., Liu T., Kong F., Yang C., Yan G., Sun L., Zhao A. (2019). Tumor-derived extracellular vesicles inhibit osteogenesis and exacerbate myeloma bone disease. Theranostics.

[57] Storti P., Bolzoni M., Donofrio G., Airoldi I., Guasco D., Toscani D., Martella E., Lazzaretti M., Mancini C., Agnelli L. (2013). Hypoxia-inducible factor (HIF)-1α suppression in myeloma cells blocks tumoral growth in vivo inhibiting angiogenesis and bone destruction. Leukemia.

[58] Aravin A.A., Sachidanandam R., Girard A., Fejes-Toth K., Hannon G.J. (2007). Developmentally Regulated piRNA Clusters Implicate MILI in Transposon Control. Science.

[59] Brennecke J., Aravin A.A., Stark A., Dus M., Kellis M., Sachidanandam R., Hannon G.J. (2007). Discrete Small RNA-Generating Loci as Master Regulators of Transposon Activity in Drosophila. Cell.

[60] Lee J.H., Jung C., Javadian-Elyaderani P., Schweyer S., Schütte D., Shoukier M., Karimi-Busheri F., Weinfeld M., Rasouli-Nia A., Hengstler J.G. (2010). Pathways of Proliferation and Antiapoptosis Driven in Breast Cancer Stem Cells by Stem Cell Protein Piwil2. Cancer Res..

[61] Cheng J., Deng H., Xiao B., Zhou H., Zhou F., Shen Z., Guo J. (2012). piR-823, a novel non-coding small RNA, demonstrates in vitro and in vivo tumor suppressive activity in human gastric cancer cells. Cancer Lett..

[62] Yan H., Wu Q.L., Sun C.Y., Ai L.S., Deng J., Zhang L., Chen L., Chu Z.-B., Tang B., Wang K. (2015). piRNA-823 contributes to tumor-igenesis by regulating de novo DNA methylation and angiogenesis in multiple myeloma. Leukemia.

[63] Ai L., Mu S., Sun C., Fan F., Yan H., Qin Y., Cui G., Wang Y., Guo T., Mei H. (2019). Myeloid-derived suppressor cells endow stem-like qualities to multiple myeloma cells by inducing piRNA-823 expression and DNMT3B activation. Mol. Cancer.

[64] De Veirman K., Van Ginderachter J.A., Lub S., De Beule N., Thielemans K., Bautmans I., Oyajobi B.O., De Bruyne E., Menu E., Lemaire M. (2015). Multiple myeloma induces Mcl-1 expression and survival of myeloid-derived suppressor cells. Oncotarget.

[65] Ramachandran I.R., Martner A., Pisklakova A., Condamine T., Chase T., Vogl T., Roth J., Gabrilovich D., Nefedova N. (2013). Myeloid-derived suppressor cell regulate growth of multiple myeloma by inhibiting T cells in bone marrow. J. Immunol..

[66] De Veirman K., Menu E., Maes K., De Beule N., De Smedt E., Maes A., Vlummens P., Fostier K., Kassambara A., Moreaux J. (2019). Myeloid-derived suppressor cells induce multiple myeloma cell survival by activating the AMPK pathway. Cancer Lett..

